# Experimental and Statistical Analysis of Saw Mill Wood Waste Composite Properties for Practical Applications

**DOI:** 10.3390/polym13224038

**Published:** 2021-11-22

**Authors:** Muhammad Usman Khan, Muhammad Abas, Sahar Noor, Bashir Salah, Waqas Saleem, Razaullah Khan

**Affiliations:** 1 Department of Industrial Engineering, University of Engineering and Technology, Peshawar 25100, Pakistan; u.khan0007@yahoo.com (M.U.K.); sahar@uetpeshawar.edu.pk (S.N.); 2 Industrial Engineering Department, College of Engineering, King Saud University, P.O. Box 800, Riyadh 11421, Saudi Arabia; 3 Department of Mechanical and Manufacturing Engineering, Institute of Technology, F91 YW50 Sligo, Ireland; saleem.waqas@itsligo.ie; 4 Department of Engineering Management, University of Engineering and Applied Sciences, Swat, Mingora 19060, Pakistan

**Keywords:** wood waste, epoxy resin, differential scanning calorimeter, thermos gravimetric analysis, taguchi method, grey relational analysis

## Abstract

The utilization of composite materials is increasing at a growing rate in almost all types of products, due to their strength-to-stiffness ratio. From this perspective, natural waste composites, i.e., wood waste composites, have also been investigated for their effective and sustainable employment. This paper deals with the application of hard and soft wood waste (i.e., acacia and cedar wood) with epoxy resin polymer to develop high strength and thermally stable wood composites. Mechanical (tensile, flexural, impact, and hardness) and thermal properties of samples are studied using Differential Scanning Calorimeter (DSC) and Thermo Gravimetric Analysis (TGA), respectively. The properties are evaluated by varying the type of wood waste and its percentage by weight. Based on the Taguchi Orthogonal Array Mixture Design, eighteen experiments are investigated. Analysis of variance (ANOVA) results show that wood waste type and wood waste content have a significant effect on all mechanical properties. From the TGA analysis, it is predicted that both types of wood waste composites exhibit similar thermal-induced degradation profiles in terms of the initial and final degradation temperatures. From the DSC results, higher glass transition temperature T_g_ is detected in 10% of the hardwood waste composite, and a reducing tendency of glass transition temperature T_g_ is observed with exceeding wood waste content. Moreover, hardwood waste at 10% demonstrated improved decomposition temperature T_d_, due to strong adhesion between waste and matrix.

## 1. Introduction

The wood waste for consumable items is getting prime attention due to stringent environmental laws and waste management issues [1]. The recycling of wood waste can be increased by developing wood waste composite products [2,3]. Presently, wood processing industries have stepped into developing different types of wood waste composite products. However, the handling of wood waste needs precautionary measures, as the solid waste could be a form of poisonous dust that causes severe allergic reactions in certain conditions [4]. Besides some health concerns, potential use of wood waste is increasing in different industries, such as furniture items, boat making, pulp and paper manufacturing, construction, handicrafts, and some high-tech manufacturing industries.

It is important to investigate the specific epoxies and polymeric materials used for wood waste composites. For instance, the use of formaldehyde resin is detrimental to the environment, due to the emission of toxic gases during composite development processes [5]. The controlled usage of synthetic adhesive is decisive to eliminate the toxic and environmental impacts [6]. Other resins, such as soy-based resin and polyurethane resin, must be considered as an alternative to toxic resins in developing wood waste composites [7]. Among different types of resins, epoxy-resin is environmentally friendly and easier to process. Functionally, this resin exhibits excellent properties due to its high cross-linked characteristics, adequate strength, low cure shrinkage, increased fatigue and impact resistance, excellent thermal properties, chemical resistance, and dimensional stability [8]. The curing of epoxy-based composites is performed by adding aliphatic amines, anhydrides, and aromatics as hardeners [9,10]. These properties help in developing molds in the desired shapes. Curing cycles of epoxy-based composites specify the grade of cure of epoxy resin and significant influence on the mechanical properties of products. Epoxy/hardener ratios and ideal curing schedules are important to attain the desired properties of composite materials [11,12,13,14,15,16].

The research on polymer-based composites has opened new avenues for polymer formulations and developing new types of composites with specific properties of choice for specialized applications [17,18,19].The naturally occurring wood waste fibers added in composite materials include wood waste [12], white rice ash husk [13], pineapple leaf fiber [14], coconut shell waste, walnut hazelnut, and sunflower husk [15,16]. The utilization of wood waste fillers in polymers improves the mechanical characteristics, for example; the use of pinecone waste in high-density polyethylene (HDPE0 at different weights (5%, 10%, 15%, and 20%) substantially improves the mechanical strength [17]. In a reported study, a hybrid polymer composite-reinforced sal and teak wood flour was investigated by varying the concentration of reinforcements [18,19]. These studies explained the behavior of glass fiber reinforced epoxy composites by examining three factors; fiber weight percentage, the curing temperature, and epoxy hardener ratios. Some research studies have also used statistical techniques to evaluate the important parameters of epoxy-based wood waste composites, such as the Taguchi method [20,21]. In these studies, researchers employed statistical techniques to determine the load-dependent strain [22]. A few publications have highlighted wood waste composite testing techniques (such as tensile, flexural, etc.) for a particular application [23,24]. Some research studies elaborated the thermal behavior of natural wood fiber composites, their thermal decomposition, and flammability. The studies have shown that the existence of natural wood fibers creates an improved insulation effect, as compared to pure polymers matrices [25]. However, natural fibers consisting of lignin, cellulose, and hemicellulose, require increased processing temperature because of their cell wall enduring decomposition [26].

Differential scanning calorimetry (DSC) and thermogravimetric analyses (TGA) are thermal analysis techniques in the characterization of crystalline and amorphous pharmaceutical materials. The Thermogravimetric Analysis monitors the variations in the mass of a sample when the temperature is changed. Normally, the hearing is supplied at a constant rate over a specified time. Differential scanning calorimetry tracks the variations in the heat flow going in and coming out of the sample when there is a change in temperature under a controlled atmosphere. While performing this test, heating and cooling cycle are ensured in an inert atmosphere. TGA and DSC techniques are employed to investigate physical and chemical characteristics of composite samples [27]. A TGA curve shows the behavior of weight reduction during thermal evaporation or chemical reaction, while the DSC curve indicates exothermic and endothermic processes. These curves are used to establish T_g_ and T_d_ [28,29] temperatures of composite samples. The reported studies have shown that exothermic peaks are observed at 270 °C and 360 °C, which were assigned to the decomposition of cellulose and hemicellulose [30]. Oliveira et al. [31] investigated the thermal properties of natural palm fibers from 20 °C to 200 °C under an inert gas atmosphere (N_2_) with a heating rate of 10 °C/min. Waseem et al. [32] investigated the tensile creep behavior of PLA material. Some researchers have explored the possibilities of using wood waste composites in high-tech applications, such as an insulation material for high temperature applications in aerospace and automobiles [33,34].

Based on the literature review, it is concluded that only limited studies are reported that deal with the utilization of hardwood and softwood waste epoxy resin composites. Moreover, this research would be novel in this perspective to investigate the mechanical and thermal stability of such composites. The authors have compiled all the reports and concluded that the available studies have covered only limited characteristics of some specific composites, for example, the usage of sundiwood waste in composite [17,35], thermal study of natural fibers composite [36], and flammability of natural fiber reinforced composite [37], etc. Considering this, the presented study focuses on hard and soft wood waste (i.e., acacia and cedar wood) reinforced with epoxy resin polymer to improve the mechanical strength and thermally stability. Mechanical and thermal tests performed on different samples include tensile, flexural, hardness, impact, differential scanning calorimeter (DSC), and thermo gravimetric analysis (TGA). Mechanical strength of samples is evaluated by varying wood waste type (at two-level i.e., acacia and cedar wood), and percentage waste weight (at three levels i.e., 10%, 20%, and 30%). Based on Taguchi orthogonal array mixture design, eighteen experiments are analyzed. Analysis of variance (ANOVA) is performed to obtain the significant factors for tensile, flexural, hardness, and impact strength.

## 2. Materials and Methods

### 2.1. Material

**Figure 1:**
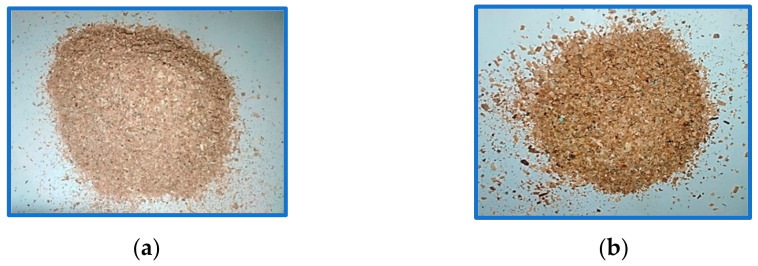
Wood waste (**a**) Acacia (**b**) Cedar.

The wood waste used in the present study is hard and soft wood, i.e., acacia and cedar. The waste of these types of wood is collected in the form of wood flour (fine powder) after sawmill machining from local manufacturing industries. The softwood and hardwood waste were filtered through 60 mesh stainless steel wire sieves that give approximately 250 microns particle size of powder. The filtered powder was then dried under the sun (with temperature variation of 35–37 °C) for 48 h. The waste (fine powder) is shown in Figure 1.

**Figure 2:**
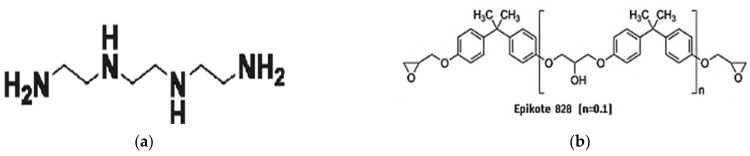
(**a**) Chemical Structure of Epikote 828 [38] (**b**) Chemical Structure of HY 951 [39].

Epikote 828 was used as epoxy resin. It is an unchanged liquid bisphenol-A–epichlorohydrin epoxide resin of medium viscosity. Epikote 828 is the standard liquid resin that is used in several applications. It provides an excellent resistance to filler settling and exhibits a great level of mechanical and chemical resistance in the final cure state. The properties of epoxy and hardener are summarized in Table 1. Figure 2a shows the structure of Epikote 828. The hardener (curing agent) used in this experimental work is HY 951 (Triethylenetetramine TETA), which is a liquid of low viscosity of an aliphatic amine. The chemical structure of HY 951 is shown in Figure 2b.

### 2.2. Sample Preparation and Responses Measurement

**Figure 3:**
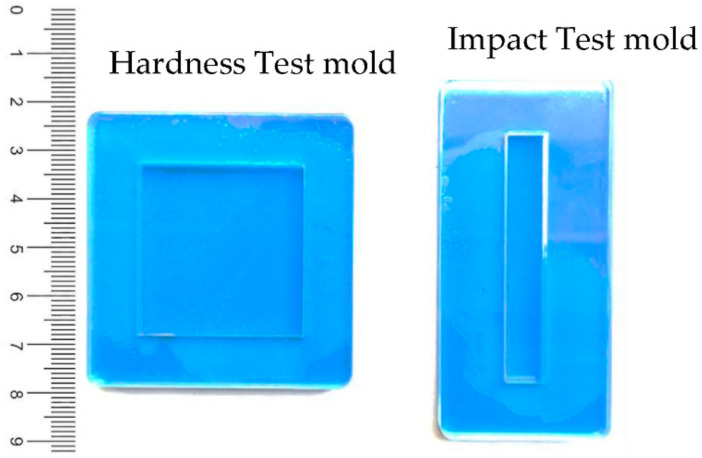
Molds for hardness and impact test, according to standard ASTM standards.

For the composite preparation, first the epoxy and hardener were mixed in a ratio of 2:1, 5:2, and 3:1 by weight [40] and mechanically stirred for approximately 5 min. Then the wood flour was mixed with solution in different weight percentages (i.e., 10%, 20%, and 30%) and mechanically stirred continuously for approximately 5 to 8 min to constitute the uniform solution. Finally, the mixture was poured into a mold/chamber and cured for approximately 24 h at room temperature. Figure 3 shows the mold designed according to ASTM standard mold for hardness and impact test.

### 2.3. Mechanical Testing

**Figure 4:**
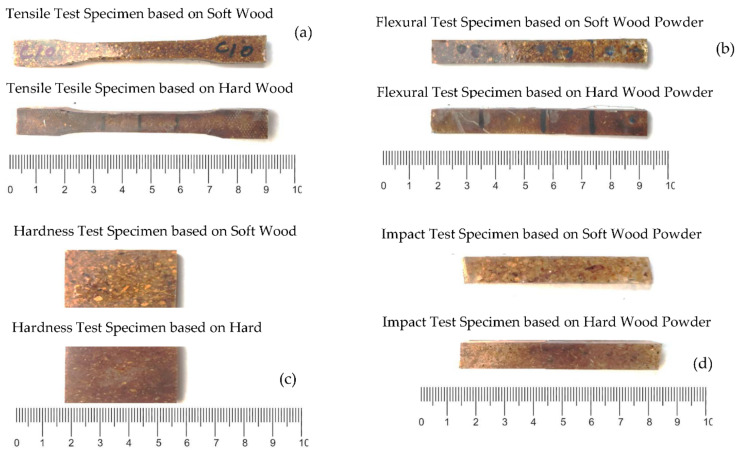
Mechanical tests (**a**) tensile test (**b**) flexural test (**c**) impact test (**d**) hardness test.

Mechanical tests, such as tensile, bending (flexural), impact, and hardness are performed to evaluate the mechanical properties of samples. Tensile, flexural, impact, and hardness tests were performed according to ASTM D638 type I standard at 2 mm/min, ASTM D790 at 1 mm/min, ASTM D4812, and ASTM D735, respectively, as shown in Figure 4.

### 2.4. Thermal Tests

Thermal properties are studied with Differential scanning Calorimetry (DSC) and Thermogravimetric Analysis (TGA). DSC is widely used as an experimental tool for a thermal investigation that detects heat flow from a specimen. Tests are performed according to a ASTM D3418-15 standard. A sample of the epoxy composite weighing approximately 3 to 10 milligrams is placed inside an aluminum hermetic pan and the aluminum capsule is sealed by means of a sample press. Finally, it is placed in a DSC machine. The heating rate at 20 °C/min is maintained to determine the conversion profile and total heat released during the dynamic curing process. The range of heat supply is monitored from room temperature to 600 °C. The data extracted are used to determine the glass transition temperature, crystallization temperature, and decomposition temperature.

The TGA is performed to study the thermal stability of a sample material as a function of its temperature. The samples were prepared according to ASTM E1131 under an inert gas atmosphere (nitrogen), and then heated slowly at a rate of 20 °C/min. TGA measures the change in weight of specimen material with respect to its function of time and function of temperature. Generally, the range of temperature is set rendering to the temperature.

### 2.5. Design of Experiments

The experiment design is an important monitoring tool for both modeling and analyzing the impact of control factors. The most important step in the experiment design is the selection of control factors and their levels. Thus, in the presented study, levels were set based on the literature review. The selected levels are shown in Table 2. Experimental runs are designed considering Taguchi L18 orthogonal array mixture design, as tabulated in Table 3. The basic purpose, in order to select the Taguchi method, is to minimize the number of experiments and cost of experimentation [41].

### 2.6. Single and Multi-Response Optimization

**Figure 5:**
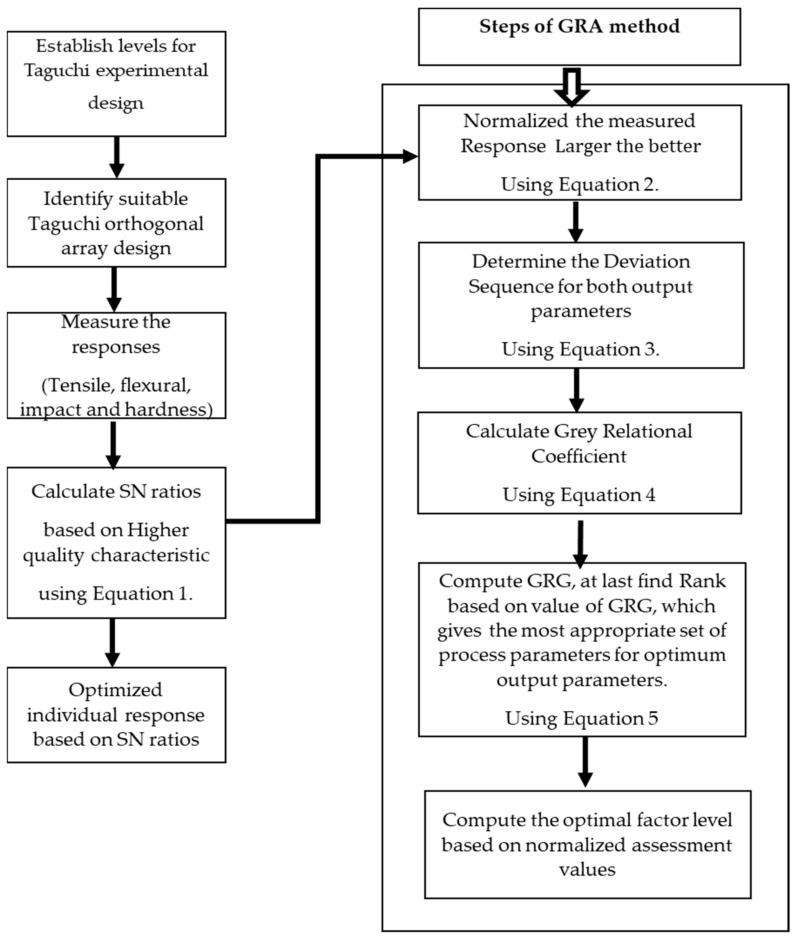
Methodology for optimization based on Signal-to-Noise ratios and Grey relational analysis.

Figure 5 shows the methodology deployed for single- and multi-response optimization based on the Taguchi signal to Noise (S/N) ratios and Grey relational analysis (GRA).

Single responses are optimized based on Taguchi S/N ratios. The Taguchi-based S/N ratios determined the variation in the quality characteristics of responses from desired values [42]. In the present study, the objective function is to maximize the responses, such as tensile strength, flexural strength, impact, and hardness. The better-quality characteristics (S/N ratios) are measured using Equation (1):

(1) $$ɳ=-10\log(\frac{1}{n}\;\overset{n}{\underset{j=1}{\sum }}\frac{1}{y_{j}^{2}})$$

where, η = SN ratio in db, *y_j_* = Experimentally observed values (*j*th experiment), *n* = no of experiment.

For multi-response optimization, GRA is deployed. The steps followed for GRA are:

*Step 1: Normalization*

The multiple responses are normalized between the range of zero and 1 by applying the formula to ignore the effect of implementing different units and to reduce the variability. The normalized equation corresponding to the higher the better can be evaluated as:

(2) $$P_{i}(j)=\frac{xi\left(j\right)-\min xi\left(j\right)}{\max xi\left(j\right)-minxi\left(j\right)}$$

*Step 2: Calculate Deviation Sequence*

By applying the normalized value of responses, Equation (3) gives the deviation sequence for a given reference and comparability sequence.

€_0i_ = ||Y_0_(j) − Y_i_(j)|| (3)

where €_0_ = deviation sequence, Y_0_(k) = max value, Y_i_(k) = current value

*Step 3: Calculate the Grey Relational Coefficient GRC:*

The GRC [43] is calculated to direct the relationship between the ideal (finest) and actual normalized experimental results. This can be expressed in the form of Equation (4):

(4) $$\xi_{i}=\frac{€min+\xi €max}{{€}_{0i}\left(j\right)+€\max\xi }$$

where €_0i_ = deviation sequence

*Step 4: Apply Grey Relational Grade GRG:*

The use of GRG [44] is considered by taking an average of GRC corresponding to each experiment. The basis of overall responses of the multiple performances characteristics is the GRG, which is considered as the prime function. Thus, optimization of complex multi-purpose characteristics is changed to a single GRG. Equation of GRG can be obtained as:

(5) $$\beta_{i}=\frac{1}{n}\sum_{j=1}^{n}\xi i\left(j\right)$$

where β_i_ = GRG for experiments, *n* = no of performance characteristics.

## 3. Results and Discussion

### 3.1. Probability Plots and Analysis of Variance (ANOVA)

**Figure 6:**
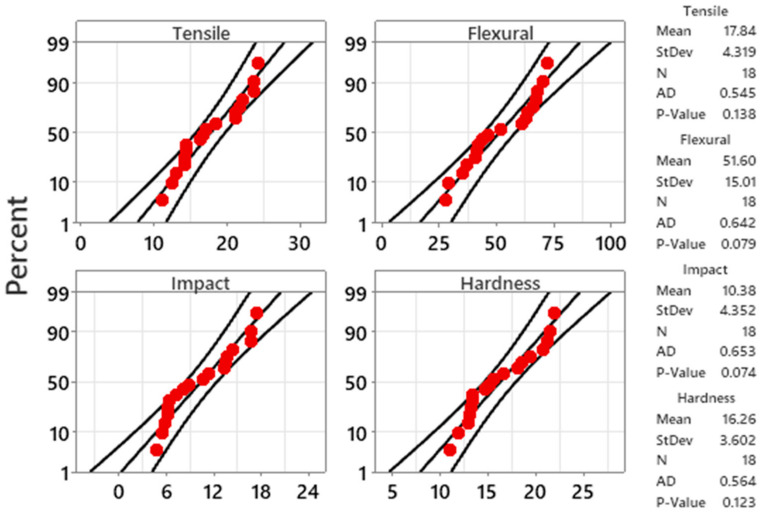
Probability plots for response. AD: Anderson–Darling Test.

The dispersal of experimental data for each response variable was examined considering the probability plots. The plots were constructed for each response variable at a 95% confidence interval, as shown in Figure 6. The results highlighted the data points for all measured values that fall near the fitted line, so it signifies that the results followed a normal distribution. However, a statistical test, namely Anderson–Darling (AD), is performed to further validate the normal distribution assumption of data. The *p*-value of the AD test greater than 0.05 represents a normal distribution of the input data. From the result, being the AD test value for each response, the *p*-value was observed greater than 0.05, which explains that the composed data follow the normal distribution and are suitable for optimization and further analysis.

ANOVA is performed at the 95% confidence level to study the effect of manufacturing process parameters on different responses for the wood waste composite. As shown in Table 4, the ANOVA illustrates that the wood waste percentage and wood waste type have a significant effect on all considered mechanical properties. The probability *p*-values are less than 0.05, while the ratio of epoxy and hardener is found insignificant, as their *p*-values are greater than 0.05.

### 3.2. Tensile Strength

**Figure 7:**
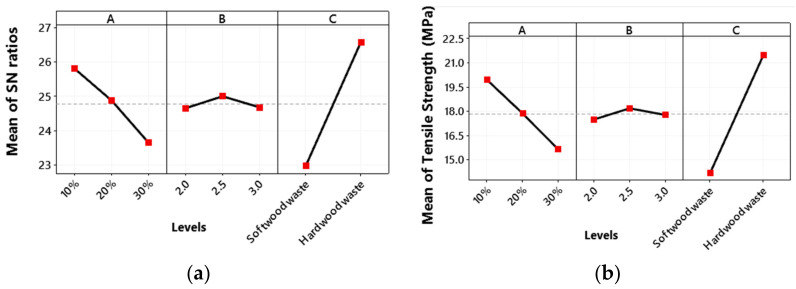
Main plot of tensile strength for (**a**) SN ratios (**b**) tensile strength means.

The main plot for the S/N ratio for under tensile loading (the larger the better) is shown in Figure 7a. The greater the value of the S/N ratio corresponds to the better the quality. The optimal combination of design parameters is the wood waste type percentage at low level, i.e., 10%, weight percentage ratio of epoxy, the hardener is at medium level i.e., 2.5, while the wood waste type is hard wood waste. Kumar et al. [35] also obtained the highest S/N ratio of the tensile load at 10% of wood waste.

The main effect plot for tensile strength (Figure 7b) shows the highest tensile strength for hard wood waste. This may be attributed to the presence of pores or vessels in hardwood that provide good adsorption capacity for resin. This results in stronger bonding between hardwood powder and polymer matrix to improve the mechanical properties of composite structure [45]. Further, the hard wood has less hydrophilic group than softwood, so the interface bonding between softwood powder and hydrophobic epoxy is weak as compared to hardwood powder [46,47]. Tensile strength decreases with an increase in the wood waste type percentage from low level 1 (10%) to a higher level 3 (30%). This may be due to a decrease in epoxy resin percentage that binds the composite firmly [48]. The other possible reason may be the weak interfacial bonding between polymer matrix and filler contents that decreases the tensile strength of the composite. According to Huda et al. [49], the accumulation of wood powder or inadequate hydrogen bonding between wood powder and epoxy resin matrix causes a decrease in tensile strength. Accumulation of wood powder creates stress concentration zones and produces early cracks that reduce the tensile strength [50]. However, with an increase in the weight percentage ratio of epoxy and hardener, the tensile strength first increases from low level 1 (2) to medium level 2 (2.5) and then decreases with a further increase in the weight percentage ratio of epoxy–hardener to a higher level of 3 (3). According to Szabelski [51], increasing the hardener ratio in epoxy resin decreases the level of polymerization and therefore reduces the adhesion with other mixing additives.

### 3.3. Flexural Strength

**Figure 8:**
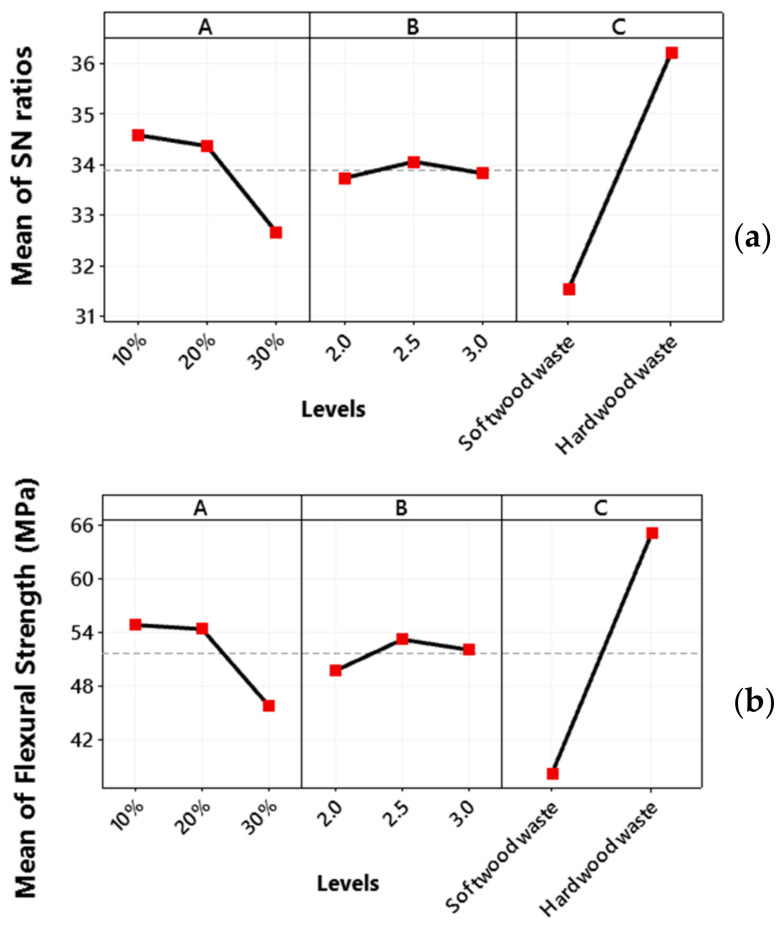
Main plot of flexural strength for (**a**) SN ratios and (**b**) flexural strength means.

Figure 8a shows the optimal combination process parameters for flexural strength, i.e., the wood waste type percentage at a low level (10%), the weight percentage ratio of epoxy hardener at a high level (3), while the wood waste type is hard wood waste.

The main effect plot for flexural strength (Figure 8b) shows the tensile strength decreases with an increase in the wood waste type percentage from a low level 1 (10%) to a high level 3 (30%). A high content of wood powder causes aggregation at various regions in the composite that result in weak bonding. Therefore, it produces cracks during flexural tests and reduces the load-bearing capability of composite [52,53]. The incapability of buttressing of wood powder to sustain the stresses transferred from polymer matrix and wood powder bonding create spaces between matrix materials and buttressing and results in a weak structure [54,55]. However, with an increase in the weight percentage ratio of epoxy and hardener, the flexural strength increases slightly from a low level 1 (2) to medium level (2.5). This decreases with an increase in weight percentage ratio of epoxy from medium level to a higher level of 3 (3).

### 3.4. Impact Strength

**Figure 9:**
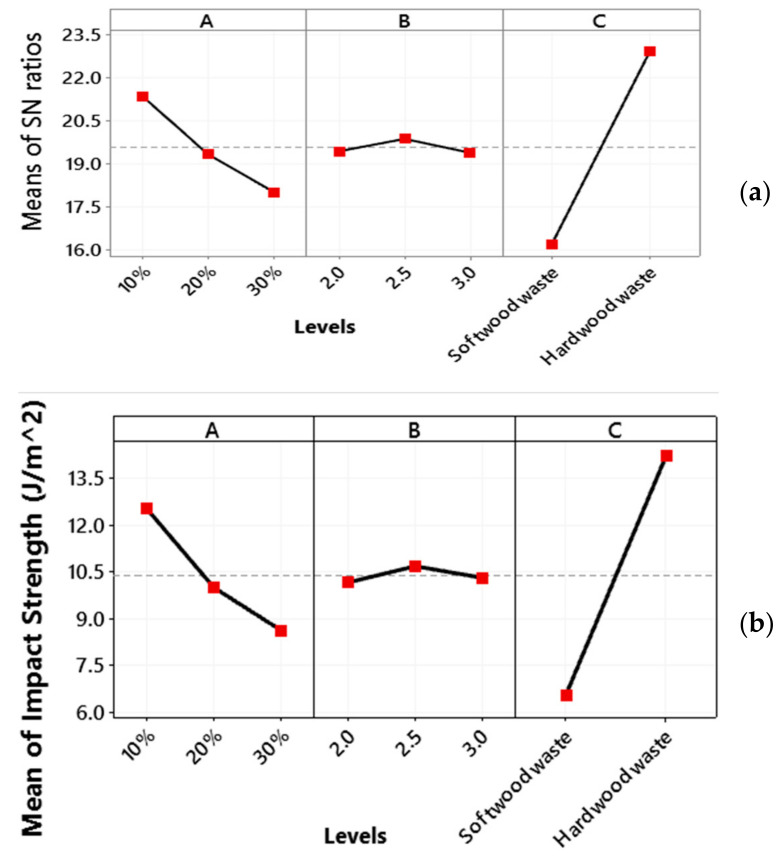
Main plot of impact strength for (**a**) SN ratios and (**b**) impact strength mean.

The main plot for the S/N ratio for impact strength is shown in Figure 9a. The optimal combination of design parameters is wood waste type percentage at a low level, i.e., 10%, the weight percentage ratio of epoxy is at medium level, i.e., 2.5, while the wood waste type is a hard wood waste.

The main effect plot for means of impact strength is shown in Figure 9b. It shows that the highest impact strength is observed for hard wood waste. Further, tensile strength decreases with an increase in the wood waste type percentage from low level (10%) to a high level (30%). This may be attributed to molecular flexibility of polymer that plays a key role in determining the relative toughness and brittleness of composites [48]. The other possible reason is the formation of micro spaces at the fiber–matrix interface with an increase in wood powder that induces the crack propagation in the structure [56,57]. An increase in poor interfacial wetting decreases the impact strength of wood powder-based composites. The increase in wood ash quantity causes a brittle fracture in composite with less impact energy [48]. With the increase in weight percentage ratio of epoxy and hardener, the impact strength first increases slightly from a low level (1) and then jumps up to a medium level of 2.

### 3.5. Hardness

**Figure 10:**
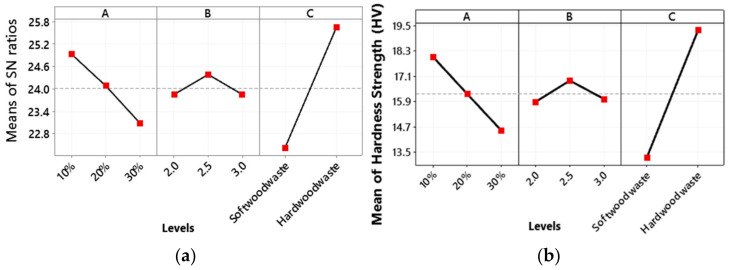
Main plot of hardness for (**a**) SN ratios and (**b**) hardness means.

Figure 10a shows the optimal combination process parameters for hardness i.e., wood waste type percentage at a low level, i.e., 10%, the weight percentage ratio of epoxy is at a medium level, i.e., 2.5, while the wood waste type is a hard wood waste.

The main effect plot for flexural strength means in Figure 10b shows that the hardness decreases with an increase in the wood waste type percentage from a low level 1 (i.e., 10%) to a high level 3 (i.e., 30%). This is attributed to a decrease in epoxy resin percentage that binds the composite firmly [48]. An increase in wood powder makes the composite structure porous and therefore reduces the hardness of material [57]. With an increase in the weight percentage ratio of epoxy and hardener, the hardness first increases slightly from a low level 1 to a medium level 2, and then decreases with an increase in weight percentage ratio of epoxy from a medium level to a high level 3. The maximum hardness is observed for hard wood waste.

### 3.6. Multi-Response Optimization Using GRA

As discussed in the material and methods section, the responses are optimized based on the GRA. First, the responses are normalized using Equation (2) to harmonize the units. These are shown in Table 5. In the second step, the value of the deviation sequence is attained by using Equation (3), as tabulated in Table 6. In the third step, the Grey Relational Coefficient (GRC) is obtained based on Equation (4). This was evaluated to choose the “larger the better” class that reflected the relationship between the desired test data and actual data, as shown in Table 7. In step 4, the GRG is computed using equation 5, as shown in Table 8. Higher GRG values correspond to the optimal combination of process parameters. Hence, the maximum value of GRG is computed as 0.781 in the 4th experimental run. This indicated the wood waste type percentage at a low level, i.e., 10%, the weight percentage ratio of epoxy hardener at a high level, i.e., 3, while the wood waste type is a hard wood waste.

### 3.7. Thermal Testing Results

#### 3.7.1. Differential Scanning Calorimetry DSC

**Figure 11:**
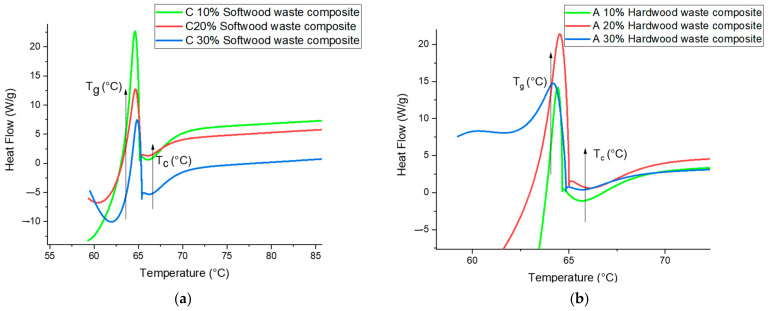
(**a**) Glass transition temperature (T_g_) and crystallization temperature T_C_ of softwood waste filled in composite. (**b**) Glass transition temperature T_g_ and crystallization temperature T_C_ of hardwood waste filled in composite.

The data evaluation calculated through DSC analysis showed optimistic results. The phase transition of hardwood and softwood waste epoxy composite can either be exothermic or endothermic. The heat flow was measured with respect to time and glass transition temperature (T_g_), and crystallization Temperature T_c_ and decomposition temperature T_d_ were observed. The glass transition temperature shows the precise temperature; however, if the temperature crosses the T_g_ then the material behaves like a viscous fluid. With the help of DSC machine, the change in temperature observed for these phases is shown in Figure 11a,b. The early peak from 60 to 70 °C specifies the T_g_ of both specimens. It is observed that T_g_ lies in between 62 to 66 °C. Finally, the highest T_g_ is detected for 10% wood waste loading. When the waste exceeds more than 10%, a decreasing tendency of T_g_ is examined. The glass transition curve exhibited a similar trend for waste wood loading, both for a hardwood and softwood waste epoxy composite. However, it is recognized that the T_g_ of polymer composite depends on the strength of the chain segment of organic compounds [58,59,60]. The glass transition curve exhibited a similar trend for waste wood loading, both for a hardwood and softwood waste epoxy composite. From basic research, due to a reduction in wood waste moisture, surface treatment of wood waste and solid interfacial bonding between wood waste and the matrix takes place, which results in improving the T_g_. The next exothermic region found around 65 to 69 °C, as shown, with slow rate of crystallization for both hardwood and softwood waste composite; however, in order to increase, the crystallization peak needs to improve the mobility of chain segment in the polymer composite [61]. The next exothermic region was found around 65 to 69 °C, as the crystallization region for both hardwood and softwood waste composite.

**Figure 12:**
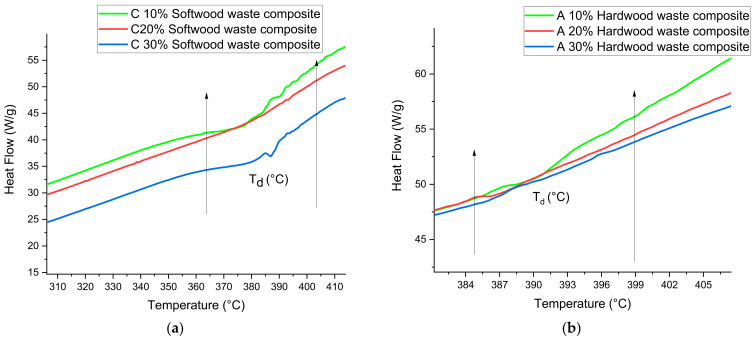
(**a**) Decomposition temperature T_d_ of softwood waste filled in composite. (**b**) Decomposition temperature T_d_ of hardwood waste filled in composite.

Figure 12a,b demonstrate the decomposition temperature T_d_ for both wood waste types composites. The T_d_ of hardwood and softwood waste composite was around 380 to 400 °C. The previous studies indicate that pure epoxy and bamboo epoxy composite exhibit dissimilar T_d_ [36]. The bamboo fiber composite and epoxy composite exhibited exothermic peaks at temperatures 327 °C to 354 °C. Hence, the addition of hardwood waste at A 10% improves the T_d_, due to its strong adhesion between matrix and waste. However, the other samples C 10%, C 20%, C 30%, A 20%, and A 30% slightly improved the value of T_d_.

#### 3.7.2. Thermogravimetric Analysis TGA

**Figure 13:**
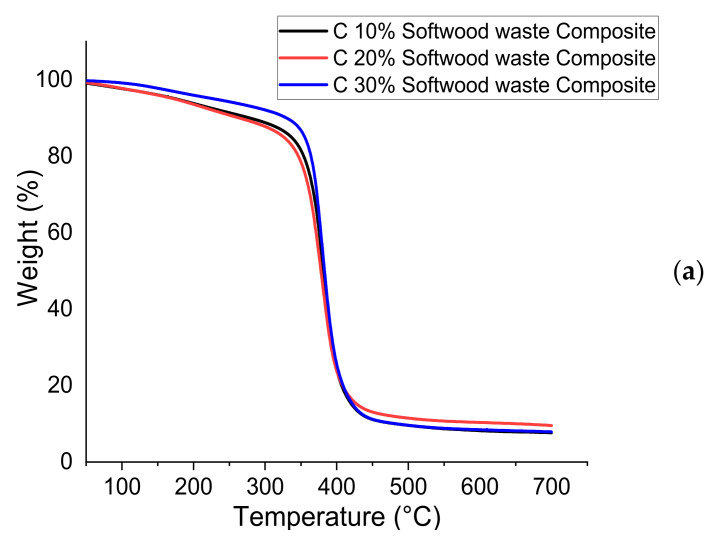

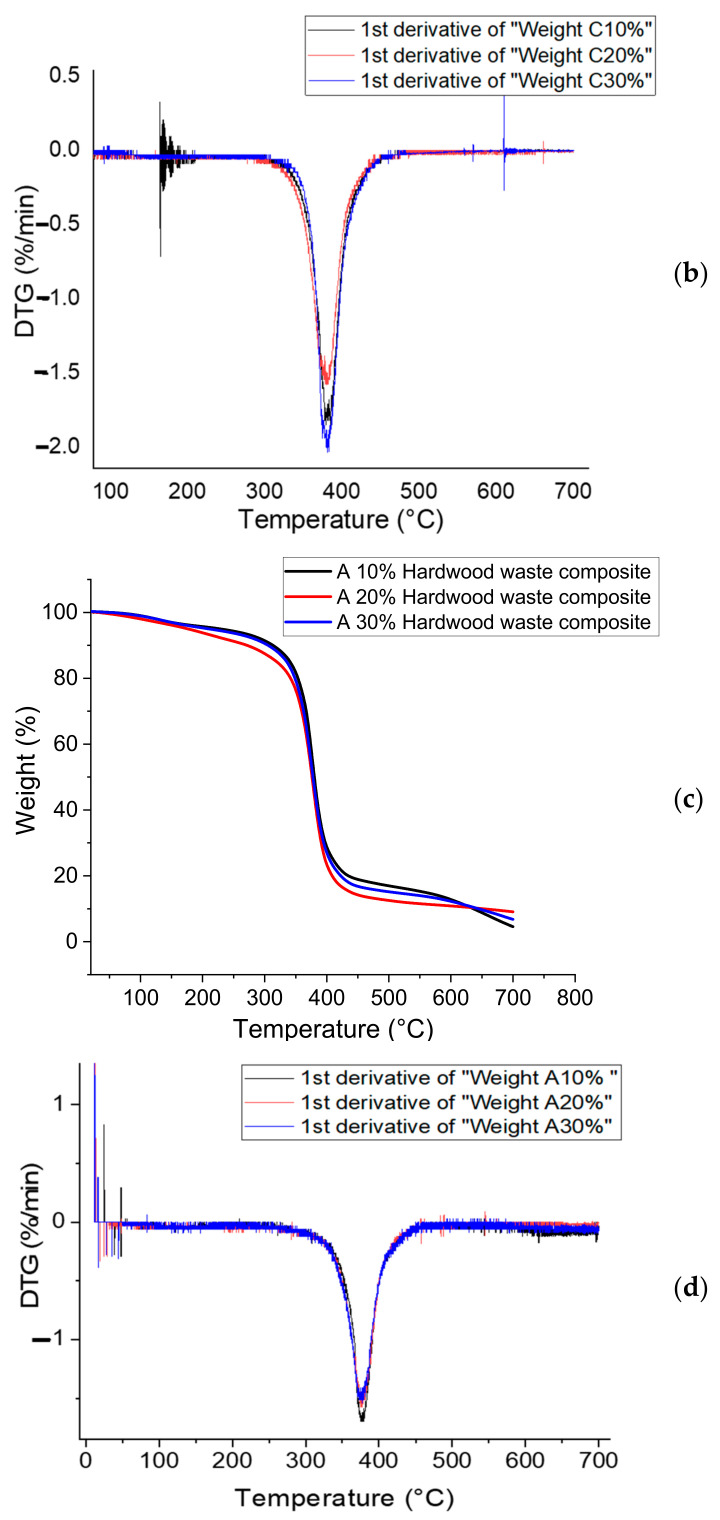
(**a**) Weight loss curves for soft wood. (**b**) Derivative of weight loss DTG for softwood waste composite. (**c**) Weight loss curves for hard wood waste. (**d**) Derivative of weight loss DTG for hardwood waste composite.

The results of applied hardwood and softwood waste on the thermal stability of epoxy composite are evaluated through TGA. The degradation of epoxy composite exists in two stages. From the first stage, the degradation takes place at 50 °C to 100 °C. This is due to the natural connection of Hemicelluloses and moisture portion [62], therefore, wood fibers existing of more Hemicelluloses content absorb further moisture and degrade at lesser temperatures. The second stage is a significant degradation stage when the organic compounds contained in the composite are decomposed; this is because of the main lignocellulose constituents [63]. However, the treatment was found to support in emitting the impurities and other low thermal content portions; different studies investigated in the chemical behavior may enhance the material thermal stability by eliminating the inoperable materials, such as non-cellulosic [64,65]. The values computed from the weight loss curves (TGA) and derivatives of weight loss (DTG) are presented in Table 9. The plots of these are shown in Figure 13a–d. The peak that appears on the DTG curves explains the maximum rate of weight loss from the specimen and generates information about its chemical structure. As a result of the increase in temperature, the dehydration occurs first, accompanied by the discharge of liquid and volatile compounds [37,66,67].

The thermal degradation of the hardwood and softwood waste epoxy composite was determined based on TGA temperature profiles corresponding to 5%, 10%, and 50% weight. The largest temperature value was recorded on hardwood waste A 10% and softwood waste composite C 30%. Regardless of the wood waste type and quantity of wood waste, the temperature of 5% weight loss was more than 150 °C, which is higher than the predictable temperature of the utilization of epoxy composites. Additionally, it was analyzed that the material shows maximum thermal stability in the temperature range around 300 °C. The higher temperature values were obtained for the C 10%, C 20%, and A 20% among the epoxy composites. Hence, the degradation characteristics of different lignocellulosic fibers are possibly assessed based on their chemical composition. Therefore, it is credible that alterations in thermal stability of composite samples from different wood species can be attributed to dissimilarities and variations in the chemical composition of timber components and additionally, can also affect the thermal performance wood polymer composites [68].

Table chart 11 with the residue at 700 °C shows an increased value in organic compounds in the proportion to the amount of lignin contained in it. This is accompanied by the presence of aromatic groups in lignin, the existence of which promotes the formation of char [69].

## 4. Conclusions

Several compositions of wood waste composites were analyzed by varying the weight ratios of epoxy and hardener. From these compositions, it can be concluded that the epoxy-based composite samples manufactured with both types of wood waste (acacia and cedar) show adequate mechanical strength and thermal performance.

From the statistical analysis, it was observed that hardwood waste in the epoxy binder has substantial impacts on the tensile strength, flexural strength, impact strength, and hardness. The thermal properties of wood waste epoxy based-composites studied with TGA and DSC characterizations predicted promising results. The results illustrate that the thermal degradation of the hardwood and softwood waste epoxy composite is prominent at the temperature values that correspond to 5%, 10%, and 50% weight loss (noted from the TGA curve). In the region I degradation (T < 150 °C), evaporation of water molecules occurs below 100 °C in acacia and cedar waste epoxy based-composites. The final degradation temperature of both types of wood waste composites was observed at 380 to 400 °C.

The phase transitions of hardwood and softwood (T_g_, T_c,_ and T_d_) were observed cautiously. It was observed that T_g_ of both wood waste composites lie between 62 and 66 °C. A higher value of the T_g_ was observed at 10% waste loading. It was also noted that an increase of hard wood waste improved the value of T_d_, due to strong adhesion between waste and matrix.

## Figures and Tables

**Table 1:** Properties of epoxy and hardener.

Properties of Epoxy (Epikote 828)				Properties of Hardener HY 951	
Sr No.	Property	Test Method	Value + Unit	Property	Value in Units
1	Epoxy Group Content	SMS 2062	5260–5420 mmol/kg	Molecular Weight	146.24
2	Viscosity at 25 °C	ASTM D445	9–14 Pas	Viscosity (Hoeppler) at 25 °C	450 mPas
3	Color	ASTM D1209	100 max Pt-Co	Specific Gravity at 25 °C	1.1–1.2 g/cm^3^
4	Epoxy Equivalent Weight		182–194 g	Color	Pale Yellow or yellow Liquid
5	Density at 25 °C	SMS1374	1.16 Kg/L	Boiling Point	284–287 °C
6	Flash Point (PMCC)	ASTM D93	>150 °C		

**Table 2:** Input factors and their levels.

Factors	Symbols	Levels		
Continuous Factor		1	2	3
Wood waste weight in percentage (WWP)	A	10%	20%	30%
Weight percent ratio of epoxy and hardener (WPE)	B	2.00 = 2:1	2.50 = 5:2	3.0 = 3:1
Wood waste type (WWT)	C	Softwood waste	Hardwood waste	

**Table 3:** Taguchi orthogonal array design (L_18_) and measured responses.

No. of Experiments	Factors			Responses			
	A	B	C	Tensile Strength (MPa)	Flexural Strength (MPa)	Impact Strength (J/m^2^)	Hardness (HV)
1	1	1	1	16.393	44.167	8.20	14.72
2	1	1	2	23.793	61.389	16.82	21.54
3	1	2	1	17.200	46.111	8.80	15.63
4	1	2	2	24.367	68.056	17.43	21.89
5	1	3	1	14.220	41.389	7.20	13.02
6	1	3	2	23.767	67.500	16.70	21.23
7	2	1	1	14.453	42.222	6.20	13.45
8	2	1	2	21.247	63.056	13.65	18.56
9	2	2	1	14.433	40.833	6.17	13.41
10	2	2	2	21.693	72.222	14.36	20.78
11	2	3	1	14.320	36.944	6.30	13.23
12	2	3	2	21.167	70.556	13.30	18.12
13	3	1	1	12.420	35.278	5.50	11.88
14	3	1	2	16.767	51.944	10.59	15.16
15	3	2	1	13.073	28.056	5.87	12.98
16	3	2	2	18.460	63.611	11.45	16.65
17	3	3	1	11.220	29.167	4.80	11.05
18	3	3	2	22.067	66.389	13.55	19.45

**Table 4:** Analysis of variance ANOVA (combined data of both data and wood waste composite).

Tensile Result		
Source	F-Value	*p*-Value
Regression	60.71	0.000
Wood percentage	34.14	0.012
Weight percent ratio of epoxy and hardener	0.15	0.708
Wood waste type	147.86	0.001
**Flexural Result**		
Source	F-Value	*p*-Value
Regression	45.66	0.024
Wood percentage	9.64	0.008
Weight percent ratio of epoxy and hardener	0.63	0.439
Wood waste type	126.70	0.003
**Impact Result**		
Source	F-Value	*p*-Value
Regression	109.07	0.000
Wood percentage	48.32	0.03
Weight percent ratio of epoxy and hardener	0.07	0.795
Wood waste type	278.81	0.043
**Hardness Result**		
Source	F-Value	*p*-Value
Regression	41.75	0.000
Wood percentage	22.90	0.034
Weight percent ratio of epoxy and hardener	0.03	0.859
Wood waste type	102.32	0.009

**Table 5:** Normalized responses.

Experiment No.	Tensile Strength	Flexural Strength	Impact Strength	Hardness
1	0.394	0.365	0.393	0.340
2	0.956	0.755	0.956	1.000
3	0.455	0.409	0.454	0.424
4	1.000	0.906	1.000	0.974
5	0.228	0.302	0.228	0.182
6	0.954	0.893	0.954	0.943
7	0.246	0.321	0.245	0.222
8	0.763	0.792	0.762	0.696
9	0.244	0.289	0.244	0.218
10	0.797	1.000	0.796	0.901
11	0.236	0.201	0.235	0.202
12	0.757	0.962	0.756	0.655
13	0.091	0.164	0.091	0.076
14	0.422	0.541	0.421	0.380
15	0.141	0.000	0.140	0.178
16	0.551	0.805	0.550	0.518
17	0.000	0.025	0.000	0.000
18	0.825	0.868	0.825	0.778

**Table 6:** Deviation sequence analysis for responses.

Experiment No.	Tensile Strength	Flexural Strength	Impact Strength	Hardness
1	0.606	0.635	0.394	0.660
2	0.044	0.245	0.956	0.000
3	0.545	0.591	0.455	0.576
4	0.000	0.094	1.000	0.026
5	0.772	0.698	0.228	0.817
6	0.046	0.107	0.954	0.057
7	0.754	0.679	0.246	0.778
8	0.237	0.208	0.763	0.304
9	0.756	0.711	0.244	0.781
10	0.203	0.000	0.797	0.098
11	0.764	0.799	0.236	0.798
12	0.243	0.038	0.757	0.345
13	0.909	0.836	0.091	0.923
14	0.578	0.459	0.422	0.619
15	0.859	1.000	0.141	0.821
16	0.449	0.195	0.551	0.481
17	1.000	0.975	0.000	1.000
18	0.175	0.132	0.825	0.222

**Table 7:** Grey relational coefficients for responses.

Experiment No.	Tensile Strength	Flexural Strength	Impact Strength	Hardness
1	0.452	0.440	0.560	0.431
2	0.920	0.671	0.343	1.000
3	0.478	0.458	0.524	0.465
4	1.000	0.841	0.333	0.951
5	0.393	0.417	0.687	0.380
6	0.916	0.824	0.344	0.898
7	0.399	0.424	0.670	0.391
8	0.678	0.707	0.396	0.622
9	0.398	0.413	0.672	0.390
10	0.711	1.000	0.386	0.836
11	0.396	0.385	0.680	0.385
12	0.673	0.930	0.398	0.592
13	0.355	0.374	0.846	0.351
14	0.464	0.521	0.542	0.447
15	0.368	0.333	0.780	0.378
16	0.527	0.719	0.476	0.510
17	0.333	0.339	1.000	0.333
18	0.741	0.791	0.377	0.693

**Table 8:** GRG and Ranks.

Experiment No.	Grey Relational Grade GRG	Ranks
1	0.471	14
2	0.733	3
3	0.481	12
4	0.781	1
5	0.469	15
6	0.746	2
7	0.471	13
8	0.601	7
9	0.468	16
10	0.733	4
11	0.461	18
12	0.648	6
13	0.482	11
14	0.494	10
15	0.465	17
16	0.558	8
17	0.501	9
18	0.651	5

**Table 9:** Weight loss temperature (TGA) at 5%, 10%, and 50%, and derivation of peak weight loss (DTG).

Sample	5% Weight Loss, °C	10% Weight Loss, °C	50% Weight Loss, °C	Derivative of Weight Loss (DTG), °C	Residual Sample Weight 700 °C
A 10%	227.6 °C	313.2 °C	379.9 °C	377 °C	4.50%
A 20%	175.9 °C	271.3 °C	376.1 °C	376 °C	9.07%
A 30%	209.5 °C	315.24 °C	383.5 °C	374 °C	6.77%
C 10%	175.2 °C	278.5 °C	381.2 °C	381 °C	7.63%
C 20%	173.1 °C	263.9 °C	377.8 °C	380 °C	9.54%
C 30%	228.6 °C	329.7 °C	382.8 °C	378 °C	7.88%

## Data Availability

The data presented in this study are available on request from the corresponding authors.
